# Patterns and Outcomes of Nonoperative Management of Abdominal Trauma With Solid Organ Injury Among the Pediatric Population

**DOI:** 10.7759/cureus.98820

**Published:** 2025-12-09

**Authors:** Mohammed H Kazkaz, Hussain A Al Ghadeer, Othman Almohammedsaleh, Simon A Kolanjian, Abdulelah A Alneamah, Mohammed A AlJumaah, Alla A Bokhamseen, ⁠Zaina N Alrawi, Tajah M Alaithan, Hassan Ammish, Waad A Aljubairah, Ahmed A Bu Amir

**Affiliations:** 1 Pediatric Surgery, King Fahad Hospital, Al Hofuf, SAU; 2 Pediatrics, Maternity and Children Hospital, Al Mubarraz, SAU; 3 Radiology, King Fahad Hospital, Al Hofuf, SAU; 4 Pediatrics, Dr. Sulaiman Al Habib Medical Group, Al Khobar, SAU; 5 Pediatric Emergency Medicine, Maternity and Children Hospital, Dammam, SAU; 6 Pediatric Surgery, King Abdulaziz University, Jeddah, SAU; 7 Pediatrics, Al-Ahsa Health Cluster, Al Hofuf, SAU; 8 General Surgery, King Fahad Hospital, Al Hofuf, SAU

**Keywords:** abdominal trauma, al ahsa, children, management, outcome, pattern, pediatric, saudi arabia, solid organ injury

## Abstract

Background

Trauma is the leading cause of mortality and long-term disability among children older than one year in the United States and globally, including Saudi Arabia. Abdominal trauma ranks as the third most common cause of trauma-related death in children and remains a significant contributor to morbidity despite advances in pediatric trauma care. Missed abdominal injuries, in particular, represent a major source of preventable morbidity and mortality.

Objective

This study aimed to assess the patterns, clinical characteristics, and outcomes of nonoperative (conservative) management (NOM) in children with solid organ abdominal trauma, with success rate as the primary outcome and transfusion requirement, injury severity, and hospital stay as secondary outcomes.

Materials and methods

A retrospective chart review was conducted at King Fahd Hospital, Hofuf, Al-Ahsa, Saudi Arabia, encompassing all pediatric patients with solid organ abdominal trauma between 2015 and 2020. Extracted variables included age, sex, mechanism, type of trauma, injured organs, severity of injury, management approach, and hospital length of stay.

Results

Ninety children met the inclusion criteria. The mean age was 7.1 ± 3.6 years, with the majority (55.6%) aged 5-10 years. Males predominated (67.8%). Blunt trauma was reported in 95.6% of cases, most commonly due to road traffic accidents (RTAs). The spleen and liver were the most frequently injured organs. NOM was successful in 94.4% of cases (n=85), while only 5.6% (n=5) required surgical intervention. Most children were discharged within one week of admission.

Conclusions

Blunt abdominal trauma, primarily from RTAs, was the predominant mechanism of injury in the pediatric population studied. The spleen and liver were the most commonly affected organs. NOM was highly effective, reinforcing its role as the standard of care for stable pediatric patients with solid organ abdominal trauma.

## Introduction

Abdominal trauma is the third leading cause of death in injured children and the primary cause of mortality when injuries go unrecognized [1]. Despite advances in pediatric trauma management, it still results in considerable morbidity, with missed abdominal injuries remaining a key source of preventable adverse outcomes [2]. Abdominal trauma remains a cause of significant morbidity in children despite advances in the care of these patients. Blunt injuries account for approximately 90% of pediatric trauma. According to a review of several trauma databases, approximately 8-12% of children suffering blunt trauma will have an intra-abdominal injury [3]. According to recent WHO global mortality data, injuries and violence remain among the leading causes of death in children and adolescents worldwide, contributing to more than 1 million deaths annually in individuals under 18 years of age [4].

Unintentional injuries represent nearly 90% of these injuries. In youngsters between the ages of 10 and 19, these injuries are the leading cause of death [5]. Over 95% of all pediatric injury deaths take place in low- and middle-income nations, where trauma kills more children over the age of five than HIV, malaria, and tuberculosis put together [6].

Children are prone to solid organ injury (SOI) due to proportionally larger vital organs, minimal subcutaneous tissue, and a lack of sufficient protective chest wall and abdominal musculature, as well as a smaller surface area to dissipate the force associated with pediatric trauma [7]. Abdominal trauma mostly showed with the liver being the most common organ injured. Nonoperative (conservative) management (NOM) is employed in over 95% of patients [8].

NOM in blunt trauma injury has been a practice accepted for quite a long time around the globe. During the past 30 years, there has been a significant shift to NOM in hemodynamically stable patients with traumatic solid organ injuries, with subsequent decreases in morbidity and mortality [9]. NOM is currently defined as "an initial non-surgical management strategy of a SOI. It consists of resuscitation and observation but may include use of endovascular, percutaneous, or endoscopic procedures" by the International Consensus Conference (ICC) in 2018 [10].

Given the limited regional data on pediatric solid organ abdominal trauma, particularly regarding real-world outcomes of NOM. Therefore, this study aimed to evaluate the patterns, clinical characteristics, and outcomes of NOM among children with solid organ abdominal trauma. The primary outcome was the overall success rate of NOM. Secondary outcomes included transfusion requirements, length of hospital stay, distribution of injury severity, associated injuries, and the need for operative intervention.

## Materials and methods

A retrospective record-based study was conducted from 2015 to 2020 at King Fahd Hospital in Hofuf, Al-Ahsa, Saudi Arabia. King Fahd Hospital is a 502-bed general hospital with a level II trauma service, where a dedicated pediatric surgery team manages pediatric trauma. Children aged 14 years or younger were included because this age threshold aligns with the pediatric admission policy and regional trauma protocols of the institution; patients aged 15-18 years are managed through the adult trauma pathway. The study included all children who presented with abdominal trauma associated with a radiologically confirmed SOI. Patients with isolated non-abdominal trauma, those transferred from other facilities, and those with incomplete medical records were excluded to avoid duplicate encounters and ensure consistency of early management.

Data collection and operational definitions

Data were collected using a pre-structured data extraction sheet developed by the research team. The sheet included predefined variables, standardized coding options, and clear operational definitions to reduce inter-rater variability. Two researchers independently reviewed and validated all data entries before transferring them into a secure Excel file (Microsoft Corp., Redmond, WA, USA).

Hemodynamic stability was defined as age-appropriate blood pressure and heart rate without the need for vasopressor support or ongoing transfusion after initial resuscitation. NOM was defined as initial treatment without laparotomy and included resuscitation, serial clinical examinations, monitoring of vital signs, repeated laboratory testing, and imaging when clinically indicated. NOM could include angiographic or radiologic interventions; however, interventional radiology services were not available at our center during the study period. Transfusion decisions followed institutional pediatric trauma guidelines, with packed red blood cells (PRBCs) administered for hemodynamic instability or hemoglobin <7-8 g/dL, unless earlier intervention was clinically indicated.

Failure of NOM was defined as the need for operative intervention due to hemodynamic deterioration, evidence of persistent bleeding, or complications requiring surgery. Imaging and injury grading: Initial imaging assessment included a focused assessment with sonography for trauma performed in the emergency department. Hemodynamically stable children underwent contrast-enhanced computed tomography (CT) of the abdomen. Repeat CT scans were obtained only when clinically indicated (e.g., a drop in hemoglobin or worsening abdominal pain). Solid organ injuries were graded using the 2018 American Association for the Surgery of Trauma Organ Injury Scale (AAST-OIS). A board-certified radiologist interpreted all CT scans. When grading was uncertain, a second review was performed jointly with the pediatric surgery team, and discrepancies were resolved by consensus. Variables and outcomes: extracted variables included age, sex, mechanism and type of trauma, organ injured, organ-specific injury grade, associated injuries, laboratory findings, Glasgow Coma Scale (when available), Injury Severity Score, need for blood transfusion, management approach (NOM), and length of hospital stay.

The primary outcome was the success rate of NOM in children with blunt solid organ injuries. Secondary outcomes included transfusion requirement, operative intervention, complications (such as bleeding, infection, or pseudoaneurysm), admission to the intensive care unit, length of hospital stay, and mortality. Outcomes were evaluated throughout the hospital admission.

Statistical analysis

Data were coded and analyzed using SPSS Statistics version 22 (IBM Corp. Released 2013. IBM SPSS Statistics for Windows, Version 22.0. Armonk, NY: IBM Corp.). All analyses were two-tailed, and a p-value < 0.05 was considered statistically significant. Missing data were handled using complete-case analysis, as the proportion of missing variables was low and randomly distributed. No data imputation was performed. Descriptive statistics were generated for all variables. Categorical variables were summarized as frequencies and percentages. Continuous variables were summarized using means, standard deviations, and medians. Although some variables (such as length of stay) demonstrated right-skewed distributions, they were presented using both mean and median values for transparency. Comparisons between groups were performed using the chi-square test or Fisher’s exact test when expected cell counts were small. Continuous variables were compared using independent t-tests or one-way analysis of variance, acknowledging the sample-size limitations in some subgroups, particularly for penetrating trauma. No multivariable analysis was conducted due to small event numbers and the risk of model overfitting.

## Results

We reviewed 90 children who had abdominal trauma with SOI. Their ages ranged from two months to 14 years, with a mean age of 7.1 ± 3.6. Fifty children (55.6%) were between five and 10 years old. Most children (67.8%; 61) were males (Table 1).

**Table 1:** Socio-demographic data of children with abdominal trauma and SOI SD: standard deviation, SOI: solid organ injury

Demographics	No	%
Age in years		
<5	26	28.90%
5-10	50	55.60%
>10	14	15.60%
Mean ± SD	7.1 ± 3.6	
Gender		
Male	61	67.80%
Female	29	32.20%

Table 2 shows the pattern and severity of abdominal trauma with SOI among the pediatric population. The vast majority of the children (95.6%; 86) had blunt injuries. Blunt injuries were the most common mechanism secondary to road traffic accidents (RTA; passenger) (53.5%), RTA (pedestrian) (30.2%), and falls from height (11.6%). As for the mechanism of penetrating injury (four cases), two cases were due to impalement, one case was due to air gun injury, and one case was due to hand injury. The most affected organs were the liver (46.7%) and spleen (30%), while 14 (15.6%) had multiple organ injuries, and three (3.3%) children had pancreatic injuries. Regarding injury severity, 14 (51.9%) of children with splenic injury had grade I/II, seven (25.9%) had grade III, and six (22.2%) had grade IV/V. Regarding liver injury severity, it was grade I/II in 20 (47.6%) cases, grade III in 13 (31%), and grade IV/V in 9 (21.4%) cases. A total of five children with kidney injury recorded grade III severity, and three (75%) of those with pancreas injury had severity of grade I/II. Contrast blush was documented in four (4.6%) children; none required operative or endovascular intervention, and all were successfully managed conservatively, consistent with reports that not all blushes mandate immediate intervention.

**Table 2:** Pattern of abdominal trauma with SOI among the pediatric population RTA: road traffic accident, SOI: solid organ injury

Pattern of injury	No	%
Type of abdominal injury		
Blunt	86	95.6%
Penetrating	4	4.4%
Mechanism of blunt injury (n=86)		
RTA (passenger)	46	53.5%
RTA (pedestrian)	26	30.2%
Fall from height	10	11.6%
Fall onto a heavy object	1	1.2%
Assault	1	1.2%
Handlebar injury	2	2.3%
Mechanism of penetrating injury (n=4)		
Air gun injury	1	25.0%
Handlbar injury	1	25.0%
Impalement	2	50.0%
Organ affected		
Liver	42	46.7%
Spleen	27	30.0%
Multiple organ involvement	14	15.6%
Penetrating injury	4	4.4%
Pancreases	3	3.3%
Grades of splenic injuries (n=27)		
Grade I/II	14	51.9%
Grade III	7	25.9%
Grade IV/V	6	22.2%
Grades of liver injuries (n=42)		
Grade I/II	20	47.6%
Grade III	13	31.0%
Grade IV/V	9	21.4%
Grades of kidney injuries (n=6)		
Grade I/II	1	16.7%
Grade III	5	83.3%
Grades of pancreatic injuries (n=4)		
Grade I/II	3	75.0%
Grade III	1	25.0%
Contrast blush		
No	81	95.2%
Yes	4	4.7%

**Figure 1:**
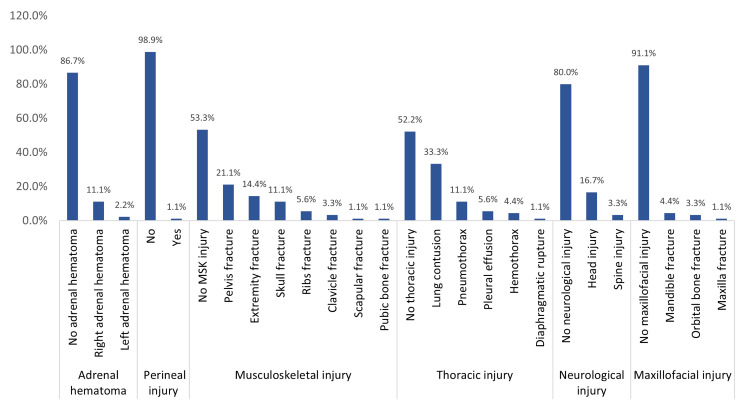
Associated injuries in pediatric patients with abdominal trauma and SOI SOI: solid organ injury

Figure 1 shows the associated injuries with abdominal trauma and SOI among the pediatric population. A total of 47.8% of the children had thoracic injuries, mainly lung contusion (33.3%) and pneumothorax (11.1%). About 46.7% had musculoskeletal injuries, mainly pelvis fracture (21.1%), extremity fracture (14.4%), and skull fracture (11.1%). Additionally, 20% had neurological injuries, mainly head injuries (16.7%) and spinal injuries (3.3%); 13.3% had adrenal hematoma; 7.9% had maxillofacial injuries, mainly mandible fractures (4.4%) and orbital fractures (3.3%); and 1.1% had perineal injuries.

Table 3 shows the laboratory findings among children with abdominal trauma with SOI. Fifty-nine (68.6%) children had abnormal aspartate aminotransferase (AST) levels, averaging 276.5 IU/L. Additionally, 61 (70.9%) had abnormal alanine aminotransferase (ALT) levels, with an average of 212.5 IU/L. Patients with proven liver injury, isolated or combined, had an average AST level of 727 and an average ALT level of 562. Amylase was abnormal among all measured cases (4 cases), with an average value of 522. The initial mean hemoglobin and hematocrit levels were 10.2 ± 2.2 g/dl and 31.0 ± 6.6, respectively. The repeated hemoglobin and hematocrit levels were 8.1 ± 2 g/dl and 26 ± 4.5, respectively.

**Table 3:** Laboratory findings among children with abdominal trauma and SOI AST: aspartate aminotransferase, ALT: alanine aminotransferase, SD: standard deviation, SOI: solid organ injury

Laboratory findings	No	%
AST (in U/L)		
Normal	27	31.40%
Abnormal	59	68.60%
Mean ± SD	495.7 ± 734.4	
Median		276.5
ALT (in U/L)		
Normal	25	29.10%
Abnormal	61	70.90%
Mean ± SD	374.6 ± 475.3	
Median		212.5
Amylase (in U/L)		
Abnormal	4	100.00%
Mean ± SD	623.8 ± 334.7	
Median		522
Hemoglobin g/dl		
Mean ± SD	10.2 ± 2.2	
Median		10.3
Hematocrit %		
Mean ± SD	31.0 ± 6.6	
Median		32.4
Repeated hemoglobin g/dl		
Mean ± SD	8.1 ± 2	
Median		8
Repeated hematocrit %		
Mean ± SD	26 ± 4.5	
Median		25

Table 4 outlines the management methods and transfusion patterns among children with abdominal trauma and SOI. A significant finding of this study is the high NOM success rate: 94.4% (85 children) were managed conservatively, while only 5.6% (five children) required laparotomy. This highlights the effectiveness and safety of NOM as the primary treatment approach for pediatric solid-organ injuries. Regarding transfusion, 31 children (34.4%) required blood products; 29 (32.2%) received PRBCs, and two (2.2%) received both PRBCs and fresh frozen plasma (FFP). Among those transfused, 23 (74.2%) required only a single transfusion episode.

**Table 4:** Management methods and transfusion among children with abdominal trauma and SOI PRBCs: packed red blood cells, FFP: fresh frozen plasma, SOI: solid organ injury

Management	No	%
Transfusion		
No transfusion	59	65.6%
PRBCs	29	32.2%
PRBCs and FFP	2	2.2%
If yes, how many times (n=31)		
One time	23	74.2%
2 times	2	6.5%
3 times	3	9.7%
8 times/more	3	9.7%
Treatment method		
Conservative	85	94.4%
Laparotomy	5	5.6%

**Figure 2:**
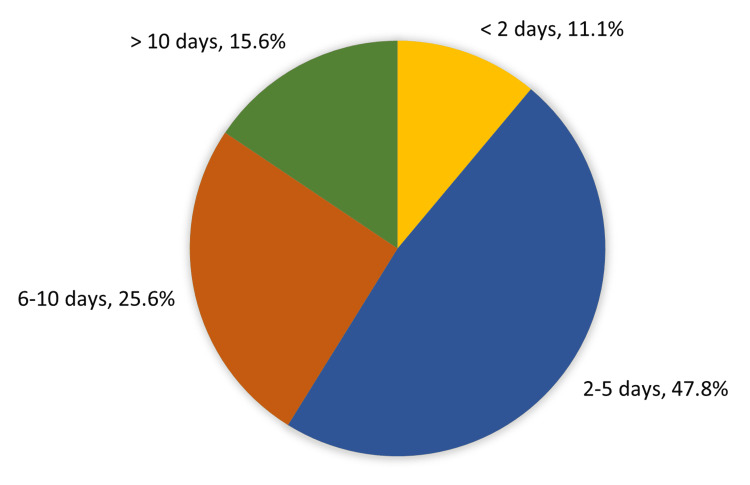
LOS (days) for children with abdominal trauma and SOI LOS: length of stay, SOI: solid organ injury

Figure 2 shows the outcome (LOS). Forty-three (47.8%) of the study cases stayed in the hospital for two to five days, 23 (25.6%) for 6-10 days, and 14 (15.6%) for more than 10 days, with a mean LOS of 6.0 ± 3.6 days.

Table 5 shows the trauma and organ injury patterns according to the type of trauma. There was no significant difference in the pattern between blunt and penetrating trauma for age, gender, or even clinical pattern and associated injuries. However, the penetrating trauma group included only four cases; therefore, these statistical comparisons should be interpreted with caution due to the very small sample size. Only the treatment method showed a significant relation with trauma type, where surgery was needed for only one case (1.2%) with blunt trauma compared to the four cases (100%) with penetrating trauma (p=0.001).

**Table 5:** Pattern of abdominal trauma with SOI among the pediatric population by trauma type P: exact probability test, * p<0.05 (significant), SOI: solid organ injury

Pattern	Mechanism of abdominal injury				p-value
	Blunt		Penetrating		
	No	%	No	%	
Age in years					0.420
<5	26	30.2%	0	0.0%	
5-10	47	54.7%	3	75.0%	
>10	13	15.1%	1	25.0%	
Gender					0.752
Male	58	67.4%	3	75.0%	
Female	28	32.6%	1	25.0%	
Organ affected					0.001*
Spleen	27	31.4%	0	0.0%	
Liver	42	48.8%	0	0.0%	
Pancreases	3	3.5%	0	0.0%	
Multiple organ involvement	14	16.3%	0	0.0%	
Penetrating injury	0	0.0%	4	100.0%	
Transfusion					0.333
No transfusion	55	64.0%	4	100.0%	
PRBCs	29	33.7%	0	0.0%	
Both of them	2	2.3%	0	0.0%	
Adrenal hematoma					0.422
No adrenal hematoma	74	86.0%	4	100.0%	
Right adrenal hematoma	12	14.0%	0	0.0%	
Perineal injury					0.828
No	85	98.8%	4	100.0%	
Yes	1	1.2%	0	0.0%	
Musculoskeletal injury					0.374
No	45	52.3%	3	75.0%	
Yes	41	47.7%	1	25.0%	
Thoracic injury					0.351
No thoracic injury	44	51.2%	3	75.0%	
Pneumothorax	42	48.8%	1	25.0%	
Neurological injury					0.593
No neurological injury	68	79.1%	4	100.0%	
Head injury	15	17.4%	0	0.0%	
Spine injury	3	3.5%	0	0.0%	
Maxillofacial injury					0.939
No maxillofacial injury	78	90.7%	4	100.0%	
Mandible fracture	4	4.7%	0	0.0%	
Orbital bone fracture	3	3.5%	0	0.0%	
Maxilla fracture	1	1.2%	0	0.0%	
Treatment method					
Conservative	85	98.8%	0	0.0%	0.001*
Laparotomy	1	1.2%	4	100%	

Table 6 shows the analysis of LOS across demographic and clinical subgroups. No significant differences were observed in LOS with respect to age group (F(2,87)=1.75, p=0.179), sex (t=−0.64, df=42.8, p=0.525), mechanism of injury (t=−1.93, df=3.5, p=0.137), presence of thoracic injury (t=−1.40, df=76.9, p=0.165), or neurological injury (F(2,83)=2.12, p=0.126). In contrast, transfusion requirement was strongly associated with a longer LOS (F(2,87)=8.68, p<0.001), with patients receiving PRBCs or combined transfusion demonstrating substantially prolonged hospitalization compared with those not requiring transfusion. Similarly, treatment modality was significantly associated with LOS, with children undergoing laparotomy experiencing longer hospitalizations than those managed conservatively (t=−2.67, df=4.9, p=0.045).

**Table 6:** Outcome and LOS among cases of abdominal trauma with SOI by injury pattern and treatment * p<0.05 (significant) LOS: length of stay, SD: standard deviation, PRBCs: packed red blood cells, FFP: fresh frozen plasma, SOI: solid organ injury

Variable	n	Mean LOS ± SD	Statistical test	Test statistic (df)	p-value
Age group			One-way ANOVA	F(2,87)=1.75	0.179
<5 years	26	4.9 ± 3.6			
5-10 years	50	6.4 ± 3.8			
>10 years	14	6.7 ± 3.0			
Gender			Welch’s t-test	t=−0.64, df=42.8	0.525
Male	61	5.8 ± 3.3			
Female	29	6.4 ± 4.5			
Mechanism			Welch’s t-test	t=−1.93, df=3.5	0.137
Blunt	86	5.9 ± 3.7			
Penetrating	4	8.8 ± 2.9			
Thoracic injury			Welch’s t-test	t=−1.40, df=76.9	0.165
No	47	5.5 ± 3.1			
Yes	43	6.6 ± 4.2			
Neurological injury			One-way ANOVA	F(2,83)=2.12	0.126
None	68	5.6 ± 3.3			
Head	15	7.7 ± 5.0			
Spine	3	6.7 ± 2.3			
Transfusion			One-way ANOVA	F(2,87)=8.68	<0.001
None	59	4.9 ± 2.8			
PRBCs	29	8.1 ± 4.4			
PRBCs + FFP	2	7.5 ± 4.9			
Treatment method			Welch’s t-test	t=−2.67, df=4.9	0.045
Conservative	85	5.8 ± 3.7			
Laparotomy	5	9.2 ± 2.7			

**Figure 3:**
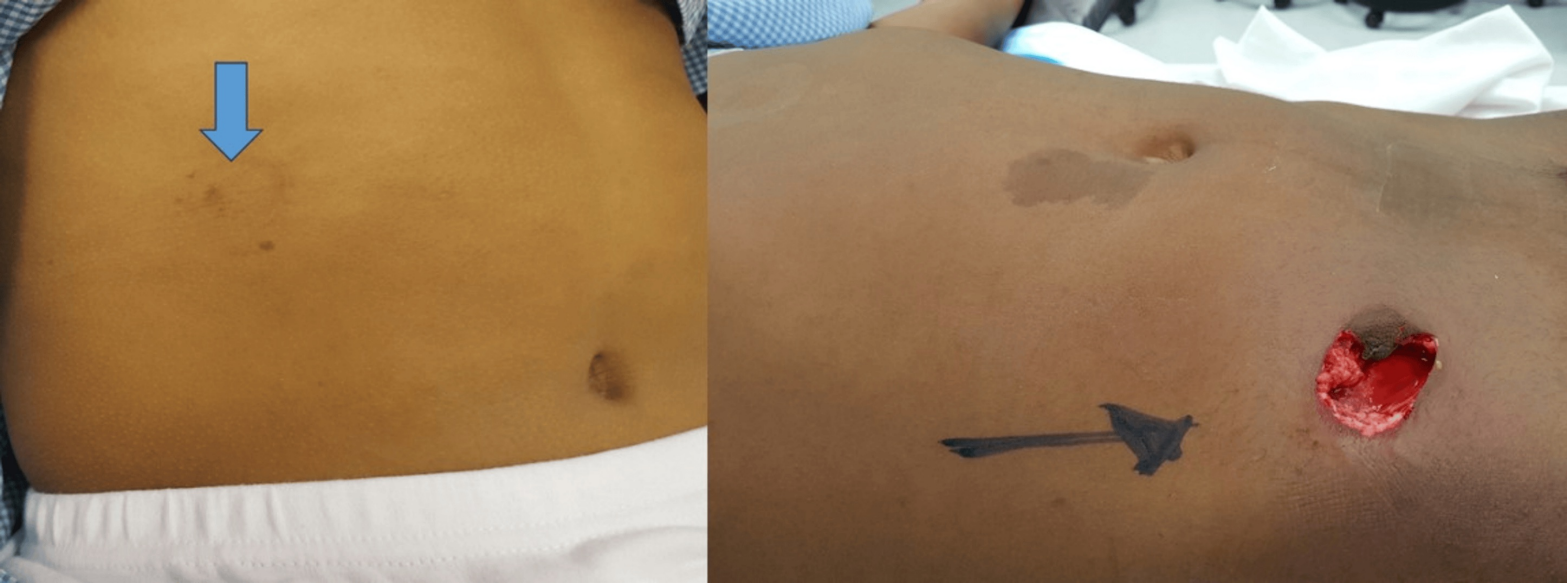
Handlebar injuries

**Figure 4:**
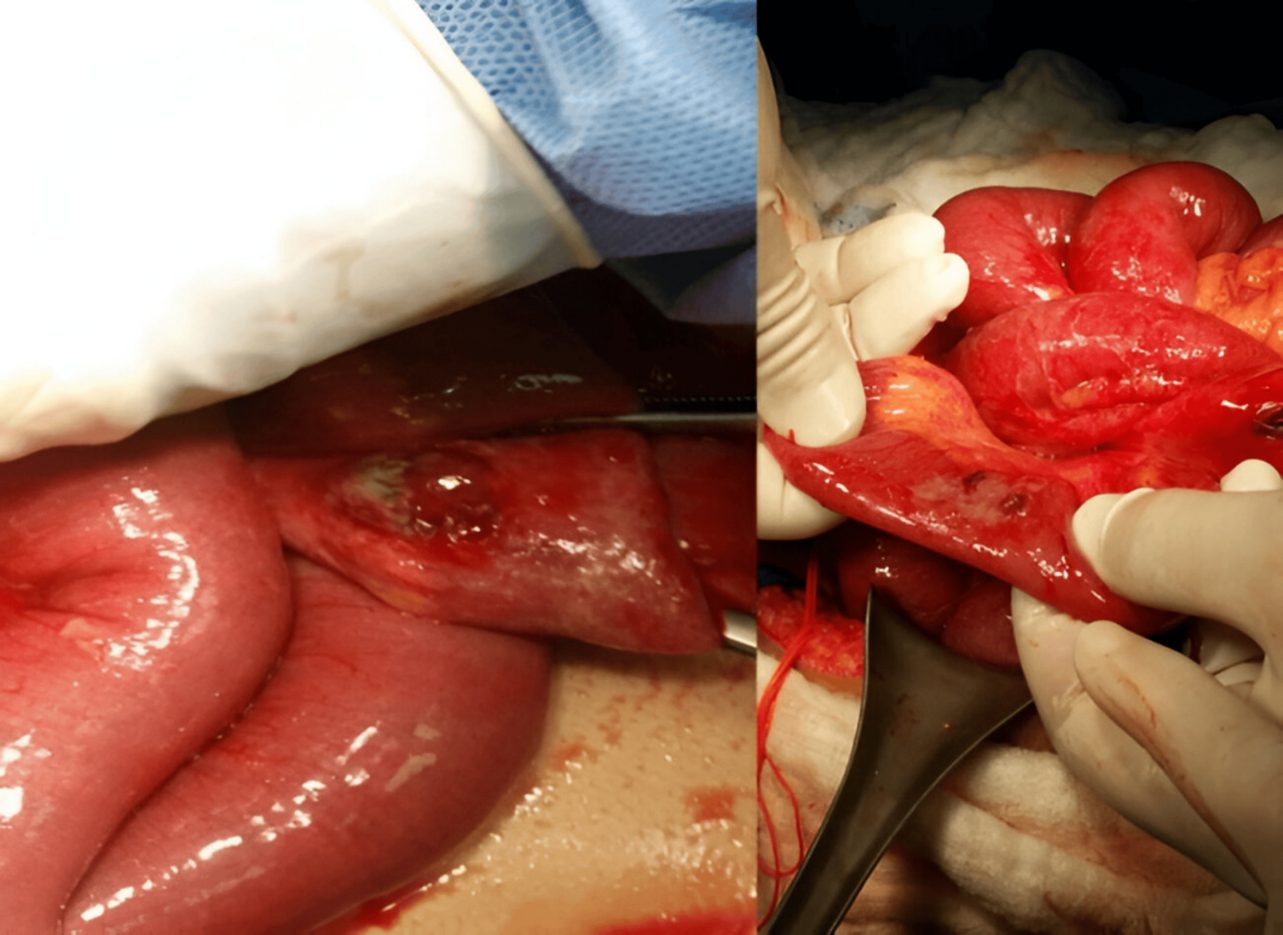
Airgun injury

**Figure 5:**
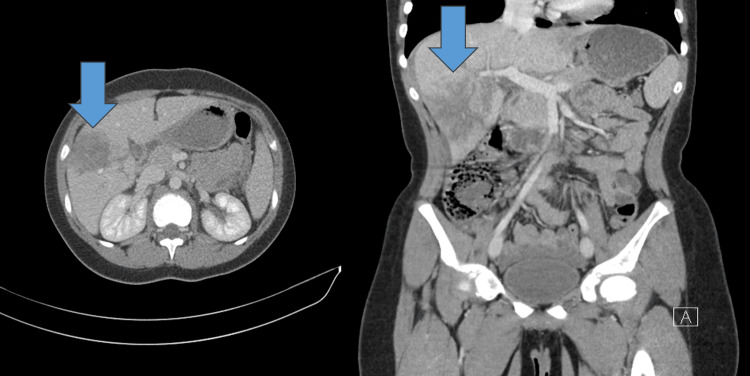
Liver injury grade III The scoring for injury grades is based on the American Association for the Surgery of Trauma Organ Injury Scale (AAST-OIS), revised in 2018.

**Figure 6:**
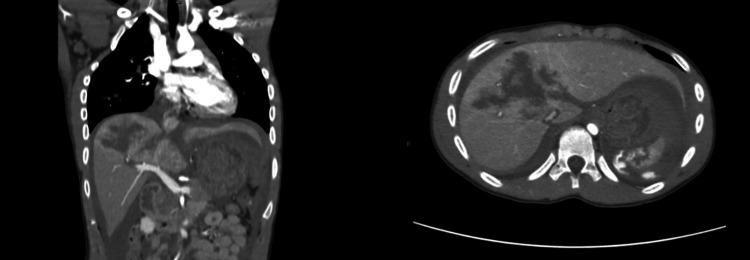
Liver grade III injury, spleen grade III injury with contrast blush, right kidney injury, and suprahepatic inferior vena cava injury The scoring for injury grades is based on the American Association for the Surgery of Trauma Organ Injury Scale (AAST-OIS), revised in 2018.

Figures 3-6 show handlebar, airgun, and solid organ injuries that were graded according to the AAST-OIS, revised in 2018.

## Discussion

In the pediatric population, trauma remains the leading cause of both morbidity and mortality. The abdomen is the third most commonly injured anatomic region in children, following only the head and extremities, and is involved in approximately 25% of major traumas. Abdominal injury can have a mortality rate of up to 8.5% and is the most common site of initially unrecognized fatal injury in traumatized children [11]. The most frequently affected abdominal organs are the spleen, liver, small intestines, and stomach, while the diaphragm is the least commonly involved. What distinguishes the present study is that it provides one of the few detailed analyses of pediatric solid organ abdominal trauma from Saudi Arabia, specifically from the Eastern region, highlighting local injury patterns, organ-specific severity, and real-world outcomes of NOM in a setting without a level I trauma center. Blunt SOI remains the predominant type worldwide; in children, the liver is most commonly injured (44%), followed by the spleen (32%), the combined liver and spleen (6%), the kidney (18%), and the pancreas (6%) [8]. The management of blunt SOI (defined as liver, spleen, or kidney injury) in children has evolved rapidly in a relatively short time. This evolution continued as increased data demonstrated satisfactory outcomes for NOM, even in high-grade injuries. With the increasing evidence, NOM of SOI in pediatric trauma is achievable in a very high percentage of patients.

In contrast to blunt abdominal trauma, penetrating injuries are significantly less common in the pediatric population compared to the adult population. Many similarities exist between the treatment of penetrating trauma in adults and children; therefore, the major tenets of adult trauma apply in the pediatric population. The current study aimed to comprehensively assess the characteristics of pediatric abdominal trauma, management in general, and especially NOM of associated SOI, and outcome with abdominal trauma. The study showed that most affected children were in the 5th to 10th age group, with an average age of 7.1 ± 3.6 years, and were mainly males. This was consistent with Basaran and Ozkan's study, in which the average age of children was 7.1 ± 4.6 years, and 58.5% of cases were male. Additionally, other studies showed a higher rate among males than females [2,12]. Male children are more active than females, with higher opportunities for outdoor playing with their colleagues, making them more liable for all types of trauma, including abdominal trauma.

Regarding the pattern of abdominal trauma with SOI, the current study showed that most of the abdominal trauma was of the blunt type, mainly due to RTA (either passenger or pedestrian), followed by a fall from a height. Basaran and Ozkan documented that of the injuries, 7.2% were penetrating traumas, and 92.8% were blunt traumas [13]. Automobile accidents accounted for 41.4% of the most frequent mechanisms of injury, which concords with the current study findings. Other studies also showed the same conclusion: More than 80% of childhood abdominal traumas are blunt traumas [7]. Traffic accidents are the most frequent cause, followed by child abuse, bicycle accidents, and falls from great heights. The liver and spleen are the organs most frequently injured [14,15]. Other studies showed that the spleen and kidneys are most commonly affected in blunt traumas, whereas the gastrointestinal tract is most commonly affected in penetrating traumas [16,17]. Kappa 2021 and [12] reported that falls were the most reported mechanism of blunt abdominal trauma among children, followed by sport-related injury and RTA [12,18].

In Saudi Arabia, Alomani et al. reported that 56 children (42%) sustained traumatic brain injury, while only 11 cases (8.3%) involved blunt abdominal trauma [12]. This contrasts with several international reports where abdominal injuries constitute a larger proportion of pediatric trauma, particularly in regions with higher rates of bicycle-related or sports-related injuries rather than motor vehicle crashes. For example, studies from Europe and North America have documented a greater representation of abdominal solid organ injuries associated with cycling accidents, sports trauma, and lower-speed mechanisms [14-17]. The relatively low proportion of blunt abdominal trauma in Alomani’s Saudi cohort and the predominance of traumatic brain injury reflect a local trauma pattern heavily influenced by high-speed RTAs, limited enforcement of child restraint systems, and infrastructural challenges that amplify head-impact injuries during crashes. In contrast, our study found a markedly higher proportion of abdominal trauma related to RTAs, consistent with trauma patterns in rapidly motorizing regions. These differences highlight how regional variations in road safety practices, vehicle restraint use, and urban development influence trauma epidemiology. Therefore, strengthening child safety measures, improving driving practices, and enhancing road infrastructure are essential steps to reduce both head injuries and abdominal trauma in the pediatric Saudi population [2,12,19-21].

Regarding SOI severity, the study showed that half of the spleen and liver injuries were moderate to severe. Still, the kidney injury was of a high severity grade. Still, kidney injury was of high severity grade in contrast to the known grade I injuries, including only parenchymal rupture and hematoma without an expansion trend, constituting 80-85% of total renal injuries [22]. It should be noted that we include only renal injuries associated with other solid organ injuries, as urologists manage isolated renal injuries. Basaran and Ozkan found that grade II injuries were the most common intra-abdominal solid injuries in children [13]. A review by Notrica reported that the median grade of injury for liver and spleen injury is grade III [14]. Other studies showed that with acute abdominal trauma, 50-70% of hepatic injuries are grades I and II, and 5% are grades IV and V. Grades I, II, III, and IV injuries were reported in a study by Leone and Hammond in 49%, 33%, 11%, and 7% [23]. On the other hand, grade I, grade II, and grades III-IV liver injuries were found in Wisner et al.'s study in 25%, 32%, and 43% of cases, respectively, with spleen injury grades of 18%, 25%, and 58%, respectively [16].

As for management, only one-third of the current study cases required transfusion, mainly blood transfusion, only once. NOM was the dominant treatment used for blunt abdominal trauma, and five children required operative management: one with blunt trauma (diaphragmatic and splenic injury) and four with penetrating trauma, consistent with the tabulated findings [24]. Notrica 2019 enlisted (1) combined liver and spleen injuries, (2) associated pancreatic injury, (3) associated renal injury, and (4) contrast extravasation on CT for failure of NON. None of them was associated with NOM failure in our study [25]. In a study by Spijkerman et al. [26], 14.9% of patients required a laparotomy, and 3.3% underwent angiographic embolization. Additionally, 5.0% of patients experienced difficulties, and 3.3% of children required intervention to address their issues. For patients receiving NOM, there was no mortality. Literature showed that surgical interventions are required among about 15% of children with intra-abdominal trauma, including hollow viscus injuries as well [17]. The current study did not report any mortality of those receiving NOM. According to Crankson, NOM was successful in over 90% of cases with a 4.7% mortality rate [27].

As for the length of hospital stay, the current study revealed that more than half of the patients stayed two to five days, one-fourth stayed six to 10 days, and a few percent stayed more than 10 days. An important observation is that we don’t have a level I trauma center with all the necessary components, especially the pediatric trauma team and trauma registry. Some important data from paramedics were missing, including patient seating and seatbelt use. We noticed a fair number of RTA victims with ejection from the vehicles, and this goes with Alghnam et al., who found that among patients injured in motor vehicle crashes, missing values on passenger seatbelts and position in the vehicle were substantially high (70.2% and 45.3%, respectively). About half of those aged 14 or older had missing information on passenger position; however, younger patients (<14 years of age) had a higher percentage of missing values for seatbelt use than older patients [28].

Overall, the findings of this study support the well-established effectiveness of NOM in hemodynamically stable children with blunt solid organ injuries, with a success rate of 94.4%. The observed patterns of injury and organ involvement are consistent with international reports, and the real-world outcomes from a level II trauma center add valuable regional evidence to the literature. However, the results should be interpreted as observational associations rather than causal effects, given the retrospective design, modest sample size, and absence of multivariable adjustment. Associations between transfusion requirements, operative management, and longer hospital stays likely reflect underlying injury severity rather than treatment effect. The penetrating trauma subgroup was too small to support generalizable conclusions; therefore, our interpretations apply primarily to blunt injury. Additionally, the lack of interventional radiology resources and center-level factors may have influenced management pathways. Despite these considerations, the study reinforces existing international evidence on the safety of NOM in most pediatric cases of blunt solid-organ injury while providing important local data from an understudied region.

Strengths

This study provides rare and region-specific data on pediatric solid organ abdominal trauma from Saudi Arabia. It offers detailed organ-specific grading, mechanism patterns, and real-world outcomes of NOM in a level II trauma setting. The high success rate of NOM aligns with international experience and adds meaningful local evidence where such data are limited.

Limitations

This study has several limitations. It is a retrospective, single-center analysis, which may limit generalizability. The small number of penetrating injuries restricted comparative statistical analyses. Although detailed clinical variables were collected, multivariable adjustment for potential confounders was not performed due to sample size constraints. Additionally, the absence of a formal trauma registry contributed to occasional missing data. Despite these limitations, the study provides important region-specific evidence in an area where pediatric abdominal trauma literature remains limited.

Our findings reinforce international evidence supporting NOM as a safe and highly effective strategy for hemodynamically stable pediatric solid organ injuries. Future multicenter prospective studies and development of standardized institutional trauma protocols may further optimize outcomes.

## Conclusions

The current study revealed that most injured children were males in their active years of movement, with blunt trauma being the predominant mechanism. The liver and spleen were the most commonly affected organs, and RTAs and falls from height were the leading causes. Most children, particularly those with blunt SOI, were successfully managed non-operatively, with an average hospital stay of less than one week. A key strength and novelty of this study is that it provides one of the few detailed assessments of pediatric solid organ abdominal trauma from the Eastern region of Saudi Arabia, offering real-world outcomes of NOM in a setting without a designated level I pediatric trauma center. These findings contribute valuable local epidemiologic data to a field where national-level pediatric trauma research remains limited. Preventive measures such as improved road safety practices, consistent use of child car seats, and supervision in outdoor environments remain essential to reduce pediatric trauma. Future research should include multicenter prospective studies with larger cohorts, establishment of unified trauma registries, and evaluation of long-term outcomes following NOM. Additionally, efforts to strengthen trauma system infrastructure, especially the development of regional pediatric trauma centers, may further improve the care and outcomes of injured children in Saudi Arabia.
